# Ancient Methods of Treatment

**Published:** 1906-10-13

**Authors:** 


### Ancient Methods of Treatment.

### OF THE CURE OF THE SCURVY.

Few diseases have brought so many bold projects to an untimely end as has the scurvy. It was the bugbear of the old navigators, whose surgeons had such ample opportunities of treatment that no apology is needed for the following description of its treatment by one who had been familiar with it from his youth upwards.

John Woodall says that on board ship "the chirurgeon ought, morning and evening, to seek for weak and poor men in their cabins, or so soon as they are missing at their messes to inquire for them, and to see their cabins be sweet and their provisions according, or to move and entreat the master or governor of the ship for redress in such cases, for fear of a general infection. And whereas the first part of this cure is in the opening of obstructions, it is therefore fit in the beginning of the grief to give a lenitive glyster; then the next day, if the party be strong, open a vein, but beware, as is said, of taking too much blood away at once, especially where the liver is weak or stopped, and where men want good nutriment, for many evils ensue thereby. The next day following his bleeding, if he can bear it, and if his disease be with a swelling or fullness, give him a dose of the pills of euphorbium or of cambogia, and make him some comfortable spoon meat, such as you can make at sea—namely, an oatmeal caudle would not be amiss of a little beer or wine, with the yolk of an egg and a little sugar, made warm and given him to drink, or any comfortable broth made with currants and other fruit, or spices moderately taken, or with sugar, or as a ship can afford; a barley water for his ordinary drink were not amiss with some few drops of cinnamon water therein, and also some juice or syrup of lemons therein, or a few drops of oil of vitriol and some sugar; and give him in his drink, by way of infusion, dried wormwood, good store, for it is very wholesome.

"Further, the chirurgeon or his mate must not fail to persuade the governor or purser in all places where they touch in the Indies and may have it to provide themselves of juice of oranges, limes, or lemons, and at Banthame of tamarinds. Also some time, though a man be well, a comfortable caudle made with some wine, spices, sugar, and the yolk of an egg were very good, for these are helps in that case as well to prevent the disease, as also to help it when it comes.

"And further experience teacheth which I have often found true, that where a disease most reigneth, even there God hath appointed the best remedies for the same grief, if it be His will they should be discovered and used; and note for substance, the lemons, limes, tamarinds, oranges, and other choice good helps in the Indies which you shall find there do far exceed any that can be carried thither from England; and yet there is a good quantity of juice of lemons sent in each ship out of England by the care of the merchants, and intended only for the relief of every poor man in his need, which is an admirable comfort to poor men in that disease: also I find that we have many good things that heal the scurvy well on land, but the sea chirurgeon shall do little at sea with them; neither will they indure (keep). The use of the juice of lemons is a precious medicine and well tried, being sound and good, let it have the chief place, for it will deserve it, the use whereof is: It is to be taken each morning, two or three spoonfuls, and fast after it two hours; and if you add one spoonful of aqua vitæ (brandy) then to a cold stomach, it is better. Also if you take a little thereof at night it is good to mix therewith some sugar, or to take of the syrup thereof is not amiss. Further note it is good to be put into each purge that you give in that disease. Some chirurgeons also give of this juice daily to the men in health as a preservative, which course is good if they have store; otherwise it were best to keep it for need. I dare not write how good a sauce it is at meat, lest the chief in the ships waste it in the great cabins to save vinegar. In want whereof use the juice of limes, oranges, or citrons, or the pulp of tamarinds; and in want of all these use oil of vitriol as many drops as may make a cup of beer, water, or rather wine if it may be had, only a very little as it were sour, to which you may also add sugar if you please or some syrups, according to your store and the necessity of that disease, for of my experience I can affirm that good oil of vitriol is an especial good medicine in the cure of the scurvy. And as touching the tamarinds brought from the Indies, they are to be eaten of themselves as the substance of them is—namely, to eat them as you would prunes: one may use this medicine so oft as he pleaseth without danger or harm, only if he fear a flux of the belly or have a weakness of the reins, let him not eat too much of the tamarinds."

John Woodall is often credited with having introduced the treatment of scurvy by giving lemon juice, but it is clear from the passages quoted above that lemon juice and lime juice were already extensively employed in the service; and it was only the weight of his name and the great popularity of his "Surgeon's Mate" that led his successors to attribute to him this great advance in treatment, just as the surgeons wrongly attributed to Ambroise Paré the method of stopping bleeding by the application of a ligature. Woodall, like Paré, would have been the first to disclaim any such originality.

Woodall was born about 1569, and, at the age of twenty, served in Lord Willoughby's expedition to render assistance to Henry IV. of France. He then travelled for many years in France, Germany, and Poland, living by the practice of his profession until his familiarity with the plague tempted him to settle in London during the great Plague year 1603. Shortly afterwards he was appointed Surgeon-General to the East India Company, and in 1616 he became surgeon to St. Bartholomew's Hospital. He was appointed in 1627 to go to Portsmouth to cure the wounded soldiers that came from the Isle of Rhé in France. He died in 1643. He is interesting to us now as the one surgeon, during the reign of James I., who carried on his craft as a profession, and not as a trade.

